# Supplementary material for: Causal Relationship between Aging and Anorexia Nervosa: A White-Matter-Microstructure-Mediated Mendelian Randomization Analysis
Source: Biomedicines. 2024 Aug 16;12(8):1874. doi: 10.3390/biomedicines12081874 (PMC11351342; doi:10.3390/biomedicines12081874)
Supplement: Supplementary file 1 [file biomedicines-12-01874-s001.zip › Supplementary Figure/Figure S4/Singe SNP plot of MR analysis for TL to ubm-b-2125.pdf]

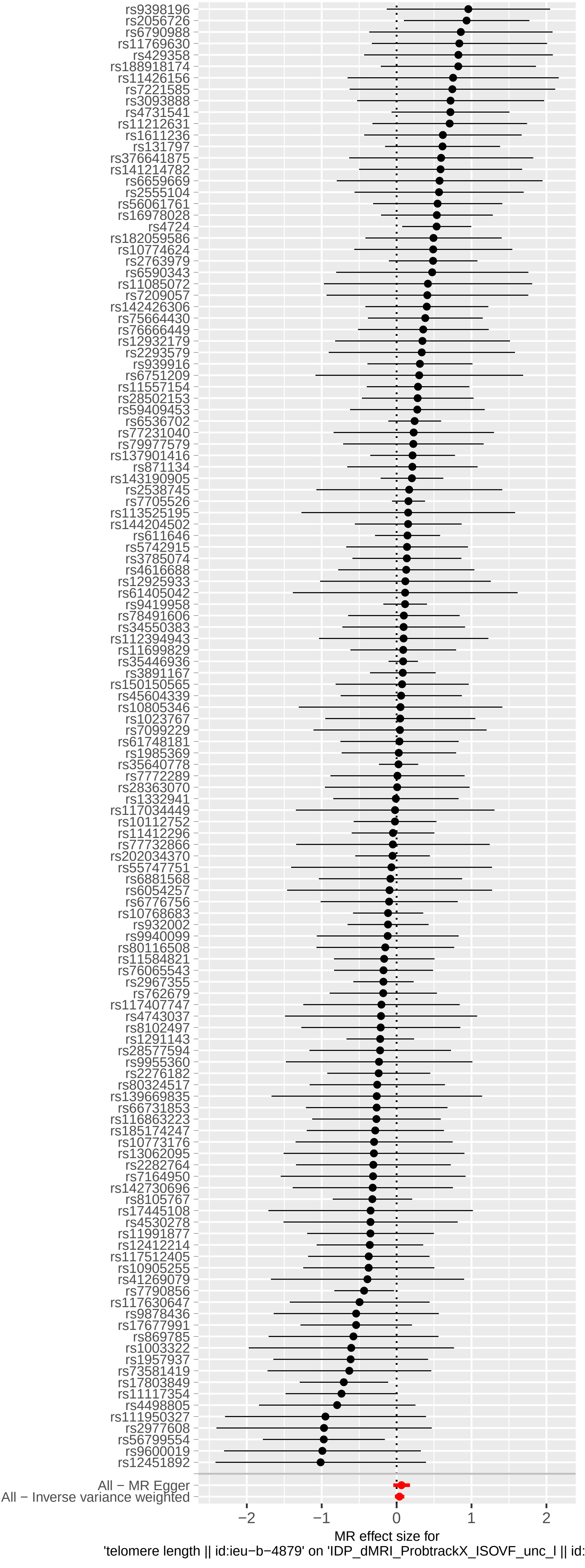
rs9398196
rs2056726
rs6790988
rs11769630
rs429358
rs188918174
rs11426156
rs7221585
rs3093888
rs4731541
rs11212631
rs1611236
rs131797
rs376641875
rs141214782
rs6659669
rs2555104
rs56061761
rs16978028
rs4724
rs182059586
rs10774624
rs2763979
rs6590343
rs11085072
rs7209057
rs142426306
rs75664430
rs76666449
rs12932179
rs2293579
rs939916
rs6751209
rs11557154
rs28502153
rs59409453
rs6536702
rs77231040
rs79977579
rs137901416
rs871134
rs143190905
rs2538745
rs7705526
rs113525195
rs144204502
rs611646
rs5742915
rs3785074
rs4616688
rs12925933
rs61405042
rs9419958
rs78491606
rs34550383
rs112394943
rs11699829
rs35446936
rs3891167
rs150150565
rs45604339
rs10805346
rs1023767
rs7099229
rs61748181
rs1985369
rs35640778
rs7772289
rs28363070
rs1332941
rs117034449
rs10112752
rs11412296
rs77732866
rs202034370
rs55747751
rs6881568
rs6054257
rs6776756
rs10768683
rs932002
rs9940099
rs80116508
rs11584821
rs76065543
rs2967355
rs762679
rs117407747
rs4743037
rs8102497
rs1291143
rs28577594
rs9955360
rs2276182
rs80324517
rs139669835
rs66731853
rs116863223
rs185174247
rs10773176
rs13062095
rs2282764
rs7164950
rs142730696
rs8105767
rs17445108
rs4530278
rs11991877
rs12412214
rs117512405
rs10905255
rs41269079
rs7790856
rs117630647
rs9878436
rs17677991
rs869785
rs1003322
rs1957937
rs73581419
rs17803849
rs11117354
rs4498805
rs111950327
rs2977608
rs56799554
rs9600019
rs12451892
All − MR Egger
All − Inverse variance weighted
−2
−1
0
1
2
MR effect size for
'telomere length || id:ieu−b−4879' on 'IDP_dMRI_ProbtrackX_ISOVF_unc_l || id:
